# The utility of administrative data for neurotrauma surveillance and prevention in Ontario, Canada

**DOI:** 10.1186/1756-0500-5-584

**Published:** 2012-10-25

**Authors:** Daria Parsons, Angela Colantonio, Michelle Mohan

**Affiliations:** 1 TorontoRehabilitation Institute, UHN, Toronto, ON, Canada; 2 Department of Occupational Science & Occupational Therapy, Toronto Rehabilitation Institute-UHN, University of Toronto, 160-500 University Ave, Toronto, ON M5G 1V7, Canada; 3 Department of Occupational Science & Occupational Therapy, University of Toronto, Toronto, Canada

**Keywords:** Prevention, Surveillance, Spinal cord injuries, Brain injuries, Data sources

## Abstract

**Background:**

Surveillance of neurotrauma events is necessary to guide the development and evaluation of effective injury prevention initiatives. The aim of this paper is to review potential sources of existing population-based data to inform neurotrauma prevention in Canada, using sources available in Ontario as an example. Data sources, including administrative data holdings from Ontario’s publicly funded health care system and ongoing national surveys, were reviewed to determine the degree of relevance for neurotrauma surveillance, using standards outlined by the World Health Organization as a framework.

**Results:**

Five key data sources were identified for neurotrauma surveillance. Five other sources were considered useful; cause of injury was not identifiable in 5 additional sources; and 4 sources were not relevant for surveillance purposes.

**Conclusions:**

We provide information about which existing data sources are most relevant for neurotrauma surveillance and research, as well as examine the strengths and limitations of these sources. Administrative data can be used to facilitate surveillance of neurotrauma and are considered both useful and cost effective for the development and evaluation of injury prevention programs.

## Background

The prevention and control of neurotrauma, including traumatic brain injury (TBI) and spinal cord injury (SCI), is important to public health. Research indicates that neurotrauma is more common than breast cancer, HIV/AIDS, and multiple sclerosis combined [1]. Although a number of prevention methods have proven effective in reducing neurotrauma, such as the use of helmets, head supports in vehicles and legislation regarding distracted and impaired drivers [2], ongoing surveillance efforts are necessary to guide the development and evaluation of effective injury prevention initiatives based on best practices. The availability of reliable data sources that capture information pertinent to neurotrauma is necessary to create effective surveillance systems.

The aim of this paper is to critically examine sources of data that can be used to identify the nature and occurrence of neurotrauma at the population level in the Canadian context, using Ontario as an example. We demonstrate the benefits of using existing data sources for surveillance by showing that data acquisition and analysis can be cost effective. The strengths and weaknesses of using administrative, survey and registry data sources for the purpose of neurotrauma prevention are also discussed.

## Method

The World Health Organization (WHO) [3] identified the following variables as the minimum necessary for effective surveillance of central nervous system injuries: Demographic (e.g., age, sex, residence); Diagnosis (e.g., ICD diagnostic codes); Cause of injury using standardized classifications such as E-codes; Circumstance of injury (e.g., locality of injury, date and time of injury, intentionality); Severity (e.g., indicated through measures such as the Glasgow Coma Scale, length of consciousness, etc.); and Outcome (e.g., as indicated through measures such as the mortality rate, discharge destination, etc.). Other information such as admission/evaluation date, discharge date, and type of care are also valuable for surveillance. Using this data profile, all publicly funded sources of data available in Ontario were reviewed to determine the extent that each presents information useful for neurotrauma surveillance. National and provincial datasets representative of the types of sources the WHO (3) considers primary for surveillance were reviewed; additional secondary sources were reviewed to determine their value as stand-alone or complementary sources. Each data source is discussed below.

## Results & discussion

**Figure 1:**
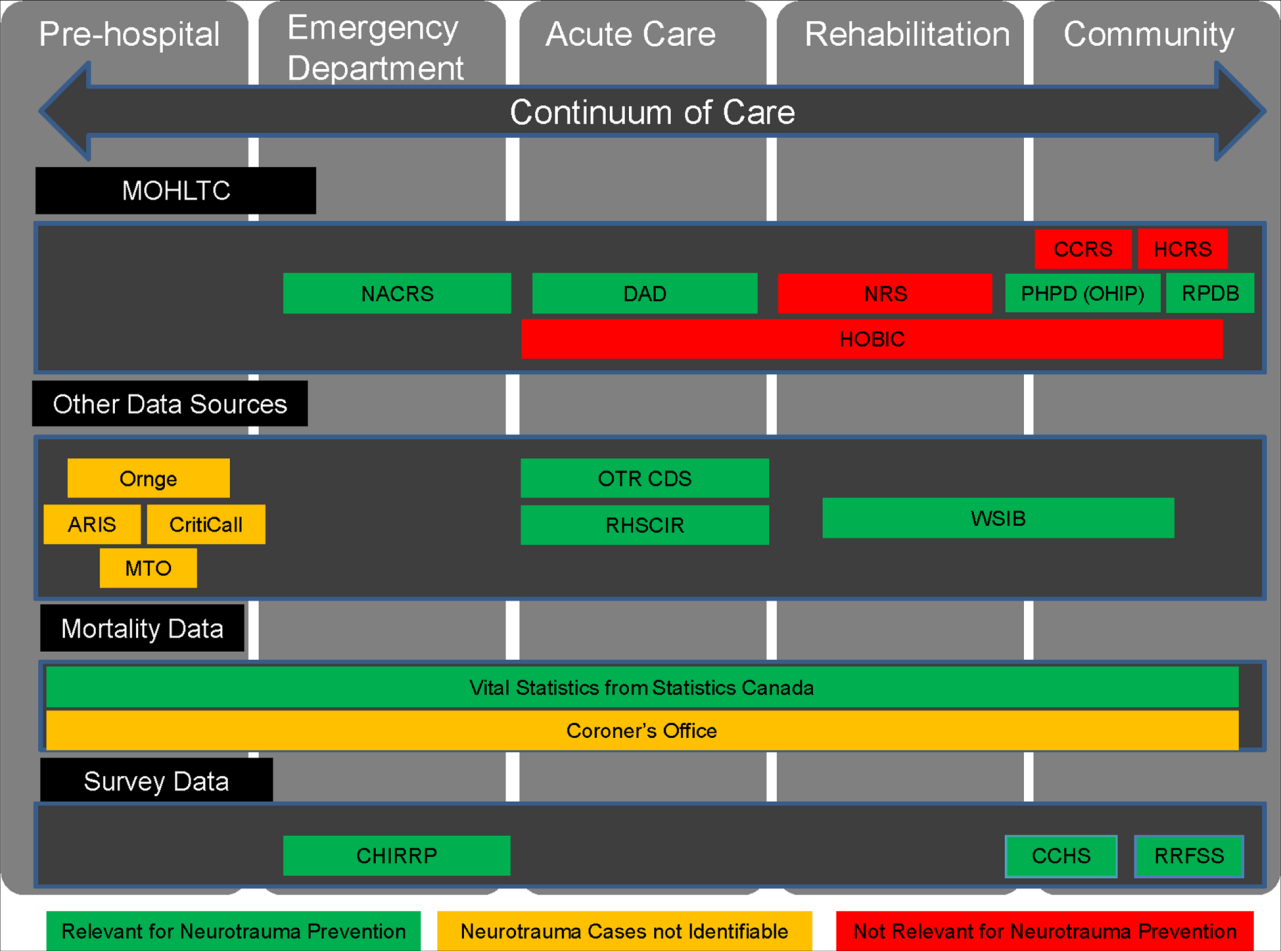
Ontario Administrative Data Sources and Relevance to Neurotrauma Prevention.

Key sources were the National Ambulatory Care Reporting System (NACRS), Discharge Abstract Database (DAD), Comprehensive Data Set of the Ontario Trauma Registry (OTR CDS), Statistics Canada’s Vital Statistics Death Database, and the Workplace Safety and Insurance Board (WSIB). Figure 1 classifies existing data sources in Ontario by degree of relevance for neurotrauma surveillance that include the WHO’s data elements.

### Data sources relevant for neurotrauma

*NACRS* includes demographic, clinical, administrative and financial information on patient visits to facility-based ambulatory care services, including emergency department visits since July 2000, for 186 participating facilities [4]. Data are collected at the time of service and submitted to the Canadian Institute for Health Information (CIHI) within 30 days of the visit. Information regarding overall severity is recorded using the Canadian Triage and Acuity Scale. The main diagnosis and intervention is coded in NACRS using ICD-10-CA codes along with up to nine additional diagnoses and interventions [4]. Data elements relevant to neurotrauma injury prevention such as Glasgow Coma Scale (GCS), helmet use and seatbelt use are included; however, completion rates for these elements are low.

*DAD*, which includes information on inpatient events from 178 acute care facilities, contains clinical, demographic and administrative data for health services provided, and hospital related outcomes (e.g. length of stay, readmissions, complications, in-hospital deaths), for each inpatient stay [5]. Up to 25 diagnoses and the mechanism of injury are coded in DAD using ICD-10-CA codes by a hospital’s health records staff, and data are electronically submitted by hospitals to CIHI monthly. Submission of records to both NACRS and DAD are mandated in the province of Ontario, thus these databases include information for individuals of all ages. Additionally, both data sources include patients’ health card number, which allows for tracking patients across the continuum of care. Postal code of the patients’ residence is also available in these databases to enable geographical analysis, so data can be reported at the county, public health unit or Local Health Integration Network level. One of the disadvantages of using NACRS or DAD is the delay in the availability of data, which can be up to eight months. Also, only in-hospital deaths are recorded; this is a severe limitation for neurotrauma prevention given that many deaths occur outside of hospital. Firsching and Woischneck [6] estimated that 68% of deaths from head injuries occurred before being admitted to hospital.

Although cases in the *OTR CDS* are included in the DAD, the advantage of the OTR CDS is the availability of data elements relevant for injury prevention surveillance (e.g., the Abbreviated Injury Scale, helmet use and seatbelt use) [7]. A limitation is that only eleven lead trauma hospitals with serious injuries (Injury Severity Score greater than 12) participate in the CDS, which means the data are based on a small subset of injury admissions.

The *vital statistics death database* includes information on all deaths and has used ICD-10 coding since 1999. This dataset includes demographics, underlying and contributing causes of death (both nature of injury and external cause), place of death, and date of death, collected from death certificates [8]. No health card number is documented, however, which limits the ability to easily link these files to other health care information. Although probabilistic linking is possible with demographic information, there is often limited access to record level data because of privacy legislation. The data are subject to the limitations relevant to data based on death certificates.

Electronic *WSIB* claims data include diagnosis for reported occupational injury cases that are insured by the WSIB. They identify neurotrauma cases beyond the hospital setting [9]; however, the quality of coding and amount of information recorded about the nature of the injury has been used to conduct research on occupational related injuries [10].

### Other relevant data sources

The *Rapid Risk Factor Surveillance System (RRFSS)*, a self-reported survey for adults used in various health units across Ontario, is intended to provide timely data for monitoring local public health issues. Participating health units assess and monitor risk behaviours, knowledge, attitudes and awareness about health-related topics, including determinants of health and health status, and provide analysis of current and emerging issues [11]. Results from RRFSS are used to facilitate program planning and evaluation set out by the Ontario Public Health Standards to advocate for public policy development and to improve community awareness of the risks for chronic diseases, infectious disease and injuries.

*The Canadian Community Health Survey (CCHS)* has a large sample size and comparisons can be made across provinces and from year to year [12]. Surveys such as the CCHS are useful to assess attitudes and behaviours related to neurotrauma or to estimate neurotrauma prevalence, particularly for mild TBIs that may not have led to an emergency room visit and/or hospitalization. Although surveys capture cases not included in hospital data, the nature of self-reported information obtained through RRFSS and CCHS is a potential limitation.

The *Rick Hansen Spinal Cord Injury Registry (RHSCIR)*[13] includes additional data on patients with SCI, and the *Canadian Hospital Injury Reporting and Prevention Program (CHIRPP)*[14] includes a sample of cases of SCI for pediatric injury-related emergency department admissions. These cases are also found in NACRS and/or DAD. The medical services data of the *Provincial Health Planning Database*[15] includes all Ontario Health Insurance Plan (OHIP) claims and can be useful to identify neurotrauma cases not identified in NACRS or DAD (i.e., individuals who did not visit the emergency department or were not admitted to acute care but saw a physician). The disadvantage is that there are limited diagnosis fields that can be used to identify neurotrauma cases.

The *Registered Persons Database* (RPDB) is useful for facilitating record linkage across datasets and to confirm deaths.

### Data sources in which neurotrauma cases are not identifiable

The *Office of the Chief Coroner of Ontario* currently utilizes a unique classification system for classifying deaths by death types, death factors and involvements rather than ICD-10-CA codes. The data collected by the coroner’s office currently includes mechanisms of injury but no diagnostic codes are available, which limits the ability to identify deaths caused by neurotrauma [16]. Diagnoses identified from manual searches of paper files containing autopsy reports include detailed injuries and have been used to inform injury prevention for neurotrauma [17]. Emergency responders such as Ornge (transport medicine including air ambulance) [18], CritiCall [19], the Ambulance Response Information System (ARIS; land ambulance) [15] and the motor vehicle collision database from the Ministry of Transportation [20] would be important sources for capturing injury data (e.g. time of injury, Glasgow Coma Scale) but currently do not include diagnosis codes identifying neurotrauma cases.

### Data sources not relevant for neurotrauma prevention

Datasets not relevant for neurotrauma prevention include sources that capture information further along the continuum of care such as the National Rehabilitation Reporting System (NRS) [21], Canadian Continuing Care Reporting System (CCRS) [22], Home Care Reporting System database (HCRS) [23], and Health Outcomes for Better Information and Care (HOBIC) [24]. These sources have limited information on mechanism of injury and other data elements relevant to prevention.

#### Practical implementation

Data from the MOHLTC— including emergency department, acute care and rehabilitation data— were analyzed to generate a neurotrauma prevention report outlining mechanisms of injury by age and sex to facilitate injury prevention planning; the researchers were also able to report on healthcare utilization for neurotrauma.

The data required for neurotrauma surveillance depend on the research question and the stage along the continuum of care under consideration; data sources often target specific points along this continuum, e.g., acute care, rehabilitation. The data source required will depend on the data elements available in a particular data source; thus, identifying and becoming familiar with all available data sources in a region is recommended. In Ontario, the MOHLTC has produced a summary of the data sources available and the data elements included in each source. If a similar resource is not available in all jurisdictions, the researcher will need to seek out this information and critically review it for its utility. In addition to death data, the emergency department, acute care, and rehabilitation administrative data were the most important sources of data for this research project.

It is difficult to objectively weigh the sources of data for neurotrauma because the value of each data source will depend on the research question. Primary data collection is exorbitantly costly so researchers should identify what data are routinely collected in their country and assess which research questions can be answered from these data; however, these data sources provide information on the direct costs of care associated with neurotrauma, which supports the need for prevention. It would be ideal to identify the research questions in advance and then collect the data elements required to address them. The province of Ontario has a rich source of administrative data available to researchers that can be used to address many of the research questions on neurotrauma prevention.

#### Availability of supporting data

The data sources discussed in this article are not available in an open source repository. The data are available from CIHI or the Ontario Ministry of Health and Long-Term Care.

## Conclusions

Developing ongoing surveillance processes and conducting epidemiologic studies to measure the impact of neurotrauma are essential components of planning and evaluating neurotrauma prevention activities. A benefit of conducting research in Ontario is the rich supply of existing data sources that can be analyzed for surveillance purposes. Using data currently being collected for other purposes (e.g., administrative data) is a cost effective, useful and sustainable way to facilitate the development of a neurotrauma surveillance system. Analyses can be conducted using these secondary data sources to identify mechanisms of injury, risk factors, preventative factors, and prevalence and incidence of neurotrauma to help inform practice and policy in Ontario.

## Abbreviations

ARIS: Ambulance reporting information system (land ambulance only); CCHS: Canadian community health survey; CCRS: Continuing care reporting system (long-tem care and complex continuing care); CHIRPP: Canadian hospital injury reporting and prevention program; DAD: Discharge abstract database; HCRS: Home care reporting system; HOBIC: Health outcomes for better information and care; MTO: Ministry of transportation; NACRS: National ambulatory care reporting system; NRS: National rehabilitation reporting system; OHIP: Ontario health insurance plan; OTR CDS: Ontario trauma registry comprehensive dataset; PHPD: Provincial health planning database; RHSCIR: Rick Hansen spinal cord injury registry; RPDB: Registered persons database; RRFSS: Rapid risk factor surveillance system; WSIB: Workplace safety and insurance board.

## Authors’ contributions

AC conceptualized the project, obtained resources for the study, supervised all aspects of the project, and participated in writing and final editing of the manuscript. DP researched content, contributed to critical analyses, writing and editing of the manuscript. MM contributed research and writing, and approved the final version of the manuscript. All authors read and approved the final manuscript.

## Competing interests

The authors declare that they have no competing interests.
